# Factors Associated with Physical Activity among People with Hypertension in a Rural Area in Bangladesh: Baseline Data from a Cluster Randomized Control Trial

**DOI:** 10.3390/ijerph18147365

**Published:** 2021-07-09

**Authors:** Fakir M Amirul Islam

**Affiliations:** 1 School of Health Sciences, Swinburne University of Technology, Hawthorn, VIC 3122, Australia; fislam@swin.edu.au; 2 Organization for Rural Community Development (ORCD), Dariapur, Narail 7500, Bangladesh

**Keywords:** rural area, Bangladesh, high blood pressure, association, logistic regression

## Abstract

The health benefits of physical activity (PA) are well recognized, and PA levels vary in different populations. The study aimed to investigate PA levels and associated sociodemographic factors among people with hypertension in a rural area in Bangladesh. Baseline data were part of a cluster randomized controlled trial of 307 adults aged 30–75 years to study the effectiveness of PA and lifestyle changes in lowering blood pressure. The outcome variables were PA at work, commuter, recreation, metabolic equivalent task (MET)-minute per week and sitting time. Total 68 (22.1%) people participated in vigorous-intensity activity, 23 (7.5%) participated in moderate-intensity sports. Overall, 83% of people were physically active more than 600 MET-min. Women (OR 2.95, 95% CI, 1.36–6.39) compared to men, and people with no education (OR 4.47, 95% CI, 1.62–12.33) compared to people with secondary school certificates or above were less physically active. Of total PA, 63% were work-related, and 1% were recreation-related for women, and these figures were 55% and 3% for men. The study reports that vigorous-intensity PA is low, and recreation time is minimal. Routine PA, especially for women and people with low education levels, should be encouraged to increase PA to manage hypertension.

## 1. Introduction

Worldwide, more than 5 million people die due to complications from being physically inactive [1,2]. The prevalence of physical inactivity is increasing irrespective of the socioeconomic condition of the countries [3], and thus, it was considered an urgent public health priority [2,4]. A 75-min vigorous-intensity PA or 150 min per week of moderate-intensity PA or combination is suggested to reduce the risk of chronic diseases [2,3].

In terms of health benefit, previous studies reported that participating in vigorous-intensity PA was associated with a reduced risk of cardiovascular diseases and mortality among adults compared to that of those participating in moderate-intensity PA [5,6,7]. Therefore, it is essential to know PA levels’ status and develop various population-specific public health awareness programs to prevent and control chronic diseases [8,9].

Bangladesh is transitioning towards a middle-income country with a significant increase in life expectancy [10], urban settlements, and economic development [11,12], which is contributing to the sedentary behaviour to rising NCDs [13,14,15]. Thus, the number of years living with numerous chronic conditions is also expected to increase, which is evident from previous studies [16,17]. However, data on the prevalence of total PA and its composition and associated factors in Bangladesh are insufficient. Two earlier studies [18,19] reported that 65% and 62% of adults aged 25 years or older were physically active, but the studies did not report the factors associated with PA. A recent survey by Moniruzzaman [20] reported the prevalence of PA and its associated factors. The study revealed that 58.1% of rural adults aged 25–64 had moderate to a high level of PA, with a significant difference between women (49%) and men (66%). Compared to these findings, studies in other low-medium-income countries reported that a high proportion of people met the world health organization recommendation for PA. For example, in Nepal, 97% of people met the world health organization recommendation for PA [21], as did Uganda (94.3%) [22], and Mozambique (90%) [23]. Previous studies conducted in low- and middle-income countries, including Bangladesh, reported that women, older age, higher education, and higher socioeconomic status were associated with a higher prevalence of physical inactivity [18,20,22,23,24,25].

Most of the previous studies conducted in Bangladesh were among the general population [18,20,26]. The patterns of PA could vary among people with a known chronic condition. Generally, people display more awareness of the risk factors of the situation and are more likely to act upon them to manage the diseases. For example, studies report that people who had diabetes or ocular diseases were more likely to be aware of the associated risk factors [27,28]. Similarly, those aware of diabetes complications were more likely to attend a diabetic retinopathy screening program [29], indicating participating in PA in people with any chronic conditions could be higher. The current study was among the people with hypertension. Previous studies reported that less than one-fifth of people with hypertension in low- to middle-income countries (LMICs) could control their blood pressure at the targeted level [30,31]. Data from the current study suggest that only one-third of the participants could maintain their blood pressure level with the cut-off of SBP ≤ 140 mmHg or DBP ≤ 90 mmHg [32], although generally, the prevalence of PA among rural people in LMICs was high [18,19,20,21,22,23,24]. Previous studies reported that participating in vigorous-intensity PA was associated with a reduced risk of cardiovascular diseases and mortality than those taking moderate-intensity PA [5,6,7]. The proportion of vigorous-intensity, moderate-intensity, or sedentary lifestyles among people with known hypertension is unknown. Findings from this study can help in conducting targeted intervention to manage blood pressure at the targeted level in LMICs. We aimed to measure the PA levels and associated sociodemographic factors among participants with known hypertension in a rural area in Bangladesh.

## 2. Materials and Methods

### 2.1. Study Sample and Location

The current study was a part of a cluster RCT that was conducted since December 2020 to study the effectiveness of PA and lifestyle changes in lowering blood pressure. The cluster RCT has consisted of 307 participants aged 30–75 years, with an equal proportion of men and women from the Banshgram Union in the Narail District. Narail is situated approximately 200 km away from the capital city Dhaka of Bangladesh. The administrative unit of Bangladesh can be briefly reported with over 163 million people divided into 64 districts. Each district divides into Upazilas (subdistricts), and each Upazila further divides into Unions which consist of 15–20 villages. Banshgram is such a Union with 18 villages [33]. The cluster RCT was reported elsewhere [34].

### 2.2. Participants

The background of the current cluster RCT is that a cross-sectional study was conducted in the Banshgram Union among 3104 participants of age 30–89 years in 2013 to report several outcomes, including the prevalence of diabetes and prediabetes [35] and known and undiagnosed hypertension. The study identified 1242 (40%) participants with hypertension [36] with a cut-off of 140/90 mmHg [37] who were the source population for the current cluster RCT. However, the current study was limit to the upper age to 75 years, leaving 1072 eligible participants for the cluster RCT from where 307 participants were recruited. Ten to twenty participants were recruited from each village based on the village size. The study location and the demographic characteristics of the source’s population were described previously [36].

### 2.3. Statistical Power

The original study is a cluster RCT with a sample size of 307 participants. This sample size was used to estimate if it had sufficient statistical power to estimate the proportion of physically active people, similar to that reported in a previous study in Bangladesh. A prior study in Bangladesh [20] reported that 60% of people aged 25–64 were physically active. If the proportion of physically active people in the current sample would be between 40–80%, the sample size was adequate with a statistical power greater than or equal to 95% and a significance level of 0.05.

### 2.4. Data Collection

Data were collected on PA, blood pressure and other variables from all participants at baseline. For the original study of a cluster randomized controlled trial, 156 participants were recruited from cluster 1, consisting of nine villages. The rest of the 307 participants were recruited from cluster 2, consisting of the rest of the villages [34]. A local nongovernment organization (NGO) in the study area in Bangladesh made the study accessible. The local investigators of the NGO conducted the recruitment along with trained data collectors. The investigators and the data collectors set up communication with the potential participants over the telephone or through direct contact and collected data from face-to-face interviews. Before data collection, the local investigators and the data collectors received training through four zoom meetings and consultation over the telephone by the chief investigator if more clarification was needed for data collection.

### 2.5. Ethics Approval and Consent Processes

Swinburne University of Technology Human Research Ethics Committee (Review reference: 20202723-5020) approved the study. The study adhered to the tenets of the Declaration of Helsinki. Written information about the project was given to each participant and was discussed their volunteer involvement in this project. The information was verbally explained to those who could not read. Upon receiving their written consents, the local investigator enrolled them in the study by assuring that participants had full rights to withdraw from the study at any stage if they wished. Participants’ decision to take part would not influence their relationship with the local NGO.

### 2.6. Participants Benefits

Participation in this study is voluntary. During the intervention, a total of 20 Omron blood pressure measuring units, one unit for each group of 15 participants, was provided to allow the monitoring of blood pressures. The machines were provided to the 20 team leaders for use by the participants and other community members. After the intervention, the blood pressure devices were not taken back, allowing the future monitoring of blood pressure in the community.

### 2.7. Measuring Physical Activity

We used the Global Physical Activity Questionnaire version 2 (GPAQ-2) [38], developed by the WHO for PA surveillance in developing countries, to measure PA levels. This questionnaire consisted of 16 questions with three domains—PA at work, PA for transportation (travel to and from places), and leisure PA, such as participating in any sports programs. Sedentary behaviour was measured by hours of sitting per day. Physical activity was categorized into vigorous- and moderate-intensity activities. Definitions of vigorous- and moderate-intensity activities and the questionnaire were described in detail in the GPAQ-2 guidelines [38]. The total PA comprised of work, commuting, and recreation activities were converted to metabolic equivalent tasks (MET) in minutes per week, which is the unit to express the intensity of PA. The methods for MET calculation were described elsewhere [18,25].

### 2.8. Outcome Measures

The outcome measures were as follows: work-related PA, including self-reported vigorous-intensity and moderate-intensity activities and their associated weekly hours spent. Commuter or travel time to and from places reported as moderate-intensity activity and weekly time spent, and recreation activity reported as vigorous-intensity sports or moderate-intensity sports and their weekly time spent, and sedentary behaviour measured as sitting time more than four hours per day. These were binary outcomes reported as “yes” for taking part in any PA and “no” for not taking part in the PA. Finally, overall PA measured as MET-min (MET-min) per week, which was classified as insufficient PA recommended by WHO with a cut-off of 0 to 600 MET-min, moderate to high PA with a cut-off of 601–6000 MET-min per week, and increased PA with a cut-off of greater than 6000 MET-min per week. Overall, PA was again reclassified below the recommended level and at least recommended level of PA.

### 2.9. Sociodemographic Factors

Sociodemographic variables included age, sex, the highest level of education, and socioeconomic status. The level of education was categorized as no schooling, primary to high school (grades 1 to 9), secondary school certificate, or above. Socioeconomic was assessed by a previously used method developed and tested in Hong Kong in terms of income insecurity and an informal labour market [39]. SES status was categorized based on the financial stability in the previous year of data collection. SES was defined as poor if participants had economic instability in the last year of data collection, middle class if they had financial instability some of the time, and wealthy if they did not have any financial instability. The participants’ occupation was categorized as a homemaker, self-managed business, farmers, and employees who include government and nongovernment employees. Self-reported diabetes status was classified as “no diabetes”, “with diabetes”, and “unknown diabetes”.

### 2.10. Statistical Analysis

Data were cleaned according to the GPAQ-2 data cleaning guidelines. In the data cleaning stage, any implausible values such as participating in any activities more than seven days a week or inconsistent answers such as days in participation were zero were checked. None of the participants gave implausible or inconsistent responses. The binary logistic regression technique was used to report the association of the sociodemographic variables with the outcome variables PA domain related to work, commuter, sitting time, and the overall PA measured by MET-min per week. The results were presented as an odds ratio with a 95% confidence interval (CI). Median and inter-quartile range (IQR) of time spent per week for work and commuter related PA were compared using the Krusical–Wallis test. Statistical software SPSS (IBM SPSS Statistics for Windows, Version 27.0.; IBM Corp.: Armonk, NY, USA) was used for the analysis.

## 3. Results

Of the total participants, the men to women ratio was equal. Half of the participants had primary to high school level of education, half of the participants were homemakers, one-third of the participants were poor, and the rest were middle class or wealthy. Fifteen per cent of the participants were 30–40 years of age, 50% were 40–59 years, and the rest were 60 to 75 years of age.

Table 1 shows the prevalence of vigorous-intensity and moderate-intensity PA at work. Of total participants, 22.1% of people participated in a vigorous-intensity activity with median (quartile 1 (Q1), quartile 3 (Q3)) 15 (6, 25) hours, and 62.2% participated in a moderate-intensity activity with median (Q1, Q3) 12 (7, 21) per week. After adjustment for age, sex, level of education, and occupation, men odds ratio (OR) (95% confidence interval (CI)) 3.44 (1.8, 6.6) compared to women, younger people such as people aged below 40 years (OR (95% CI) 7.63 (2.71, 21.5)) compared to people aged 60 years or older, and being poor was associated with a higher proportion of participating in a vigorous-intensity activity. Homemakers, employees and businesspersons were associated with a lower proportion of participating in a vigorous-intensity activity than farmers. Sex, level of education and socioeconomic condition were not associated with moderate-intensity activity at work.

Table 2 shows PA levels related to travel and recreation and sedentary lifestyle measured by sitting time more than 4 h per day. Physical activity related to travel, 77.2% of people spent more than 10 min travelling to and from places with median (Q1, Q3) 7 (7, 14) hours per week. Men OR (95% CI) 3.84 (1.94, 7.61) compared to women, people aged 40 years or younger OR (95% CI) 3.75 (1.16, 12.1) compared to those aged 60 years or older were associated with a higher proportion of PA related to travel. People with no education or employees were associated with a lower proportion of time spending more than 10 min travelling to and from places.

In terms of PA related to recreation, 7.5% of people participated in a moderate-intensity recreational activity with a median (Q1, Q3) 5 (2, 10) hours per week, only 3 (1%) people participated in a vigorous-intensity sports program. In terms of sedentary behaviour, people aged 60 years or older OR (95% CI) 4.71 (2.0, 11.1) compared to people aged 30–40 years, and people with diabetes OR (95% CI) 2.43 (1.16, 5.10) compared to people without diabetes were associated with a higher proportion of sitting time more than 4 h per day.

**Figure 1:**
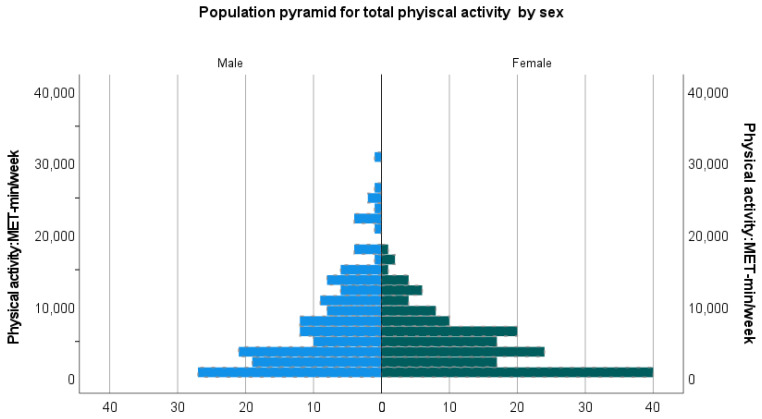
Total physical activity: MET-min per week by sex.

Overall, PA measured by metabolic equivalent task (MET)-min per week is shown in Figure 1 and Table 3. Overall, the median (Q1, Q3) MET-min was 4560 (1680, 8880). Figure 1 shows the population pyramid for total PA by sex. Men had zero to a maximum of 30,000 and median (Q1, Q3) 5520 (2400, 10,560) MET-min per week. This result was significantly higher than that in women. Women had zero to a maximum of 20,000 and median (Q1, Q3) 3720 (840, 6720) MET-min per week. Overall, 83% of people were physically active, as recommended by WHO. Of them, more than 6000 MET-min per week 40.7% and 600 to 6000 MET-min per week 42.3%. Women OR (95% CI) 2.95 (1.36, 6.39) compared to men, people aged 70 years or older OR (95% CI) 3.08 (1.49, 6.39) compared to people aged 40–59 years, no education OR (95% CI) 4.47 (1.62, 12.33) compared to people with SSC or above education levels, and employees OR (95% CI) 3.67 (1.37, 9.79) compared to farmers were physically inactive. None of those who were below the age of 40 years or businesspersons were physically inactive.

**Figure 2:**
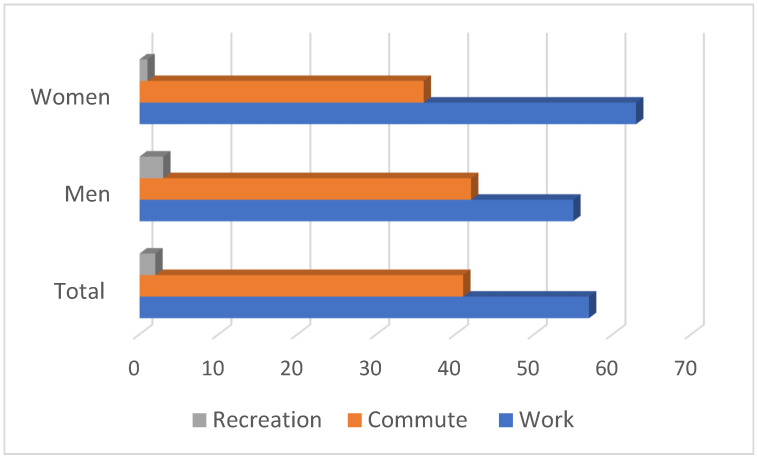
Composition of total physical activity in study sample.

The composition of total PA by sex is shown in Figure 2. Of the total who met WHO recommended guidelines, 63%, 36%, and 1% were contributed by work-related activity, commuter, and recreational activities, respectively for women, and that contributions were 55%, 42%, and 3% for men with no significant difference between sex in any of the domains’ contributions to the total PA.

## 4. Discussion

In this study, among participants with hypertension, we estimated the proportion of physically active people, time of active hours per week, sedentary behaviour, and the sociodemographic factors associated with the PA. The study also reported the composition of total PA. The significant findings from the current research include: (1) that more than one fifth of people participated in vigorous-intensity PA, more than three-quarter of people spent more than 10 min per day to travel to and from places that accounted for moderate-intensity activity; (2) based on all three domains of PA, more than 80% of rural people with hypertension were found to be physically active; (3) the significant factors associated with higher proportion of PA were male gender, and younger people, people with higher education, and people who were involved in laborious activities; (4) among the participants who were found to be active below the WHO recommended level, the proportion of women, older people, and the employees were found to be higher in this group, (5) and that of total PA, one-third was related to commute, a very minimum percentage was from any recreational activities, and the remaining proportion was work-related PA.

The current study found that more than 80% of people with hypertension were physically active, 57% of this was work-related, 41% commuter, and 2% from sports. Moniruzzaman [20] conducted a study among the general population and reported that 61% of rural adults aged 25–64 years were moderate to a high level of physically active, 77% of this was related to work, 21% related to commuter, and 2% on sports. The significant difference, especially in the source of PA, could be due to the age difference between the two studies. The current study was conducted on people aged 30 to 75 years, where one-third of the participants were more than 60 years of age compared to 9% who were 55–64 years in the previous study. The higher proportion of work-related PA in younger people could be attributed to their involvement in regular work than those of the current study. In the case of recreational activities, both studies reported the same proportion. Another study [18] conducted in a national representative survey in Bangladesh on a large sample in 2010 reported that 68.4% of people had moderate-to-high PA levels; 61% of this was related to work, 30% related to commuter, and 9% was associated with leisure PA. The proportions of leisure PA in a recent study [20] and that in our study were similar. Both studies reported a lower proportion of leisure PA among women. However, the higher proportion of leisure PA in another study is difficult to interpret. Still, it could be due to a time effect as the previous study was conducted more than ten years ago. Overall, the higher proportion of PA of the present study compared to previous studies conducted in Bangladesh [18,19,40] could be partly due to the characteristics of the study population. Previous studies reported that people with disease conditions are more aware of their risk factors and cautious about their prevention than the general population [27,28].

The current study’s prevalence of PA levels was similar to that reported in the east and southeast Asian countries ranging between 15.7–23.9, and sub-Saharan Africa ranging between 15.1–20.5 [41]. A wide variation in PA levels across different parts of the world and within the same country was previously reported [24,40,42,43]. In the current study, women, older people, and people with higher socioeconomic status were more likely to be physically inactive. These findings are consistent with previous studies in Bangladesh [18,19,20,41] and many low- and middle-income countries [24,25,40,42,43,44,45]. Irrespective of sex and age, the minimal contribution to the total PA was recreational activity, and the significant contribution was work-related physical activities. The results are also consistent with low- middle-income countries [20,24,25,42,45]. Previous studies [18,19,20,22,41,46] reported that homemakers were associated with a low level of PA, which we also found in our current study.

In contrast to previous studies [18,19,20,41,47] that reported association of higher education with a low level of PA, the current research found higher education was associated with a higher level of PA, which is consistent with some other studies [22,46]. Association of higher education with a higher PA level could be attributed to increased awareness of health benefit due to PA. Previous studies reported that people were more likely to aware of risk factors and health benefits when they had any health problems [27,28] and acted upon preventing the condition [29]. People with a low level of education were associated with a higher proportion of vigorous-intensity activity but a lower proportion of moderate-intensity PA. A higher proportion of moderate-intensity PA level among educated people might indicate that educated people were aware of overall PA. However, possibly due to the nature of their occupations, their participation in vigorous-intensity activity was low. In the current study, farmers were more likely to participate in vigorous-intensity activity than housemakers, employees, or businesspersons, which is the nature of the occupation. Although the results were not significant, employees and businesspersons were more likely to participate in recreational activities. However, recreational activities reported to be minimally contributed to the total PA in Bangladesh and other low-medium-income countries [20,24,25,42,45].

To achieve the most benefit, especially in poor resource settings, there is a need to identify factors associated with different PA levels and types. Most of the previous studies conducted in Bangladesh reported higher education associated with a low level of PA. However, the current research suggests that not higher education but some occupations such as employees and businesspersons were associated with a significantly lower level of PA, especially vigorous-intensity activity. Our study provides further insight into the factors related to PA among people with hypertension in a rural area in Bangladesh. Managing hypertension by changing lifestyle could be the best impactful practice to supplement one of the significant barriers in the health system: the shortage of qualified doctors, especially in rural areas [48,49].

The study has several strengths; for example, data were collected through face-to-face interviews that eliminate possible reporting bias. The study consists of 50% of women that present the current PA level equally by both genders. However, the research is not free from limitations, such as data being collected from a single rural location, which restricts generalization at the national level. However, the rural residents and the health structure are very comparable in Bangladesh [50].

## 5. Conclusions

Most of the participants met the WHO recommended PA level, and this is predominantly moderate-intensity activity. The PA related to recreation was very minimal. Women, older people, people with no education, and employees were associated with a low overall PA level. Routine PA should be encouraged and organized to increase vigorous-intensity activity and recreational activities to manage high blood pressure.

## Figures and Tables

**Table 1:** Activity at work and its associated sociodemographic factors in people with high blood pressures in a rural area in Bangladesh.

		Activity at Work (Yes/No of Participants) %, Weekly Hours Spent, Median (IQR)					
		Vigorous-Intensity Activity			* Moderate-Intensity		
Factors	No of Participants	*n* (%)	OR (95% CI) ^‡^	Median (IQR) ^†^	*n* (%)	OR (95% CI) ^‡^	Median (IQR) ^†^
Total	307	68 (22.1)		15 (6, 25)	191 (62.2)		12 (7, 21)
Sex							
Female	154	26 (16.9)	1.00 (reference)	11 (6, 16)	96 (62.3)	1.00 (reference)	14 (7, 21)
Male	153	42 (27.5)	3.44 (1.80, 6.61)	18 (8, 30)	95 (62.1)	1.05 (0.61, 1.83)	12 (7, 21)
Age group, years							
Below 40	46	15 (32.6)	7.63 (2.71, 21.5)	9 (6, 20)	38 (82.6)	4.08 (1.61, 10.4)	15 (8, 25)
40–59	160	42 (26.3)	4.03 (1.84, 8.83)	15 (8, 28)	107 (66.9)	2.23 (1.27, 3.9)	12 (6, 21)
60 or older	101	11 (10.9)	1.00 (reference)	11 (4, 23)	46 (45.5)	1.00 (reference)	12 (7, 16)
Level of Education							
No education	99	21 (21.2)	2.48 (0.89, 6.88)	18 (6, 35)	52 (52.5)	0.48 (0.21, 1.12)	10 (6, 18)
Primary to high school	148	36 (24.3)	2.05 (0.85, 4.93)	15 (6, 23)	96 (64.9)	0.61 (0.28, 1.30)	13 (7, 21)
SSC or above	59	11 (18.6)	1.00 (reference)	9 (4, 12)	43 (72.9)	1.0 (reference)	14 (8, 28)
Socioeconomic status							
Poor	92	27 (29.3)	1.88 (1.01, 3.49)	15 (6, 30)	58 (63.0)	1.25 (0.72, 2.17)	12 (7, 20)
Middle class	214	41 (19.2)	1.00 (reference)	14 (6, 21)	132 (61.7)	1.00 (reference)	12 (7, 21)
Occupation							
Farmer	66	28 (42.4)	1.00 (reference)	20 (12, 30) ^a^	46 (69.7)	1.00 (reference)	10 (6, 18)
Housewife	146	26 (17.8)	0.24 (0.07, 0.89)	11 (6, 16) ^a^	94 (64.4)	1.24 (0.17, 9.01)	14 (6, 21)
Employees	53	5 (9.4)	0.19 (0.06, 0.66)	9 (9, 10) ^a^	24 (45.3)	0.31 (0.12, 0.81)	10 (8, 15)
Business	24	5 (20.8)	0.27 (0.08, 0.91)	6 (4, 35) ^b^	15 (62.5)	0.42 (0.14, 1.27)	14 (8, 30)
Diabetes Status							
No diabetes	217	45 (20.7)	1.00 (reference)	13 (6, 21) ^a^	129 (59.4)	1.00 (reference)	14 (8, 21) ^a^
Diabetes	41	6 (14.6)	0.73 (0.27, 1.97)	27 (14, 35) ^b^	21 (51.2)	0.63 (0.31, 1.29)	7 (5, 12) ^b^
Unknown	49	17 (34.7)	1.63 (0.79, 3.39)	15 (6, 21) ^a^	41 (83.7)	2.74 (1.19, 6.33)	10 (7, 20) ^a^

* 51 participants did either vigorous or moderate-intensity work; ^†^ without any subscript or subscript with the same letter, e.g., “a” are for the nonsignificant difference. Different letters “a” and “b” indicates a significant difference at least at a 5% level of significance; ^‡^ OR (95% CI) adjusted for variables in model.

**Table 2:** Travel to and from places, recreational activities, and sedentary lifestyle in people with high blood pressure in a rural area in Bangladesh.

		Physical Activity (Yes/No of Participants) %, Weekly/Daily Hours Spent, Median (IQR)							
		Travel to and from Places for More Than 10 min Per Day (Weekly Hours)			Moderate-Intensity Sports (Weekly Hours)			Sitting Time ≥ 4 h Per Day	
Factors	No. of Participants	*n* (%)	OR (95% CI) ^‡^	Median (IQR) ^†^	*n* (%)	OR (95% CI) ^‡^	Median (IQR) ^†^	*n* (%)	OR (95% CI) ^‡^
Total	307	237 (77)		7 (4, 14)	23 (7.5)		5 (2, 10)	143(47)	
Sex									
Female	154	103 (67)	1.00 (reference)	6 (3, 12)	5 (3.2)	1.00 (reference)	4 (1, 8)	70 (46)	0.83 (0.49, 1.41)
Male	153	134 (88)	3.84 (1.94, 7.61)	7 (4, 14)	18 (12)	4.95 (1.55, 15.8)	4 (2, 10)	73 (48)	1.00 (reference)
Age group, years									
Below 40	46	41 (89)	3.75 (1.16, 12.1)	7 (4, 14)	10 (22)	6.74 (1.7, 26.8)	4 (2, 7)	12 (26)	1.00 (reference)
40—59	160	123 (77)	1.86 (0.95, 3.64)	7 (4, 14)	8 (5.0)	1.44 (0.43, 4.8)	6 (1, 9)	66 (41)	1.83 (0.84, 3.97)
60+	101	73 (72)	1.00 (reference)	7 (4, 14)	5 (5.0)	1.00 (reference)	7 (2,14)	65 (64)	4.71 (2.0, 11.08)
Level of education									
No education	99	67 (68)	0.25 (0.07, 0.93)	7 (4, 14) ^a^	5 (5.1)	0.67 (0.15, 3.05)	8 (4, 10)	52 (53)	1.12 (0.50, 2.50)
Primary to high school	148	114 (77)	0.31 (0.09, 1.11)	8 (5, 14) ^b^	9 (6.1)	0.67 (0.19, 2.32)	6 (1, 12)	63 (43)	0.91 (0.71, 1.15)
SSC or above	59	56 (95)	1.00 (reference)	6 (4, 10) ^a^	9 (15)	1.0 (reference)	4 (2, 4)	27 (46)	1.00 (reference)
Socioeconomic status									
Poor	92	71 (77)	1.3 (0.69, 2.44)	7 (4, 12)	9 (9.8)	2.78 (1.0, 7.85)	9 (3, 12)	38 (41)	1.00 (reference)
Middle class	214	165 (77)	1.00 (reference)	7 (4, 14)	13 (6.1)	1.00 (reference)	4 (1, 6)	105(49)	1.52 (0.89, 2.6)
Occupation									
Farmer	66	61 (92)	1.00 (reference)	8 (6, 14)	4 (6.1)	1.00 (reference)	5 (1, 10)	32 (49)	1.00 (reference)
Housewife	146	101 (69)	1.06 (0.13, 8.86)	7 (3, 12)	5 (3.4)	1.21 (0.20, 6.32)	4 (1, 8)	65 (45)	0.79 (0.13, 4.73)
Employees	53	38 (72)	0.23 (0.06, 0.82)	7 (4, 14)	3 (5.7)	1.31 (0.21, 7.95)	2 (1, 4)	32 (60)	1.18 (0.49, 2.86)
Business	24	23 (96)	1.13 (0.12, 10.4)	7 (4, 14)	5 (21)	2.98 (0.55, 16.1)	9 (6, 14)	5 (21)	0.29 (0.09, 0.95)
Diabetes status									
No diabetes	217	162 (75)	1.00 (reference)	7 (4, 14)	17 (7.8)	1.00 (reference)	5 (2, 8)	99 (46)	1.00 (reference)
Diabetes	41	34 (83)	1.45 (0.57, 3.68)	7 (4, 14)	1 (2.4)	0.28 (0.03, 2.33)	10	27 (66)	2.43 (1.16, 5.10)
Unknown	49	41 (84)	1.15 (0.48, 2.78)	8 (5, 14)	5 (10)	0.93 (0.27, 3.19)	1 (1, 4)	17 (35)	0.85 (0.43, 1.68)

^†^ without any subscript or subscript with same letter, e.g., “a” are for the nonsignificant difference. Different letters “a” and “b” indicates a significant difference at least at a 5% level of significance; ^‡^ OR (95% CI) adjusted for variables in model.

**Table 3:** Metabolic equivalent task and its associated sociodemographic factors in people with high blood pressure in a rural area in Bangladesh.

		Physical Activity (Yes/No of Participants) %, Metabolic Equivalent Task (MET)-Minute Per Week				
	No. of Participants	MET-min	High, >6000 MET-min	Moderate 600–6000 MET-min	Above vs. below Recommended Level of MET, MET-min ≥ 600 vs. MET-min < 600	
Factors		Median (IQR) ^†^	*n* (%)	*n* (%)	*n* (%)	OR (95% CI) ^‡^
Total	307	4560 (1680, 8880)	125 (40.7)	130 (42.3)	52 (16.9)	
Sex						
Female	154	3720 (840, 6720) ^a^	52 (33.8)	69 (44.8)	33 (21.4)	2.95 (1.36, 6.39)
Male	153	5520 (2400, 10,560) ^b^	73 (47.7)	61 (39.9)	19 (12.4)	1.00 (reference)
Age group, years						
Below 40	46	6960 (3360, 10,320) ^a^	26 (56.5)	20 (43.5)	--	-
40–59	160	5520 (2520, 9460) ^a^	75 (46.9)	63 (39.4)	22 (13.8)	1.00 (reference)
60 or older	101	2400 (240, 5520) ^b^	24 (23.8)	47 (46.5)	30 (29.7)	3.08 (1.49, 6.39)
Level of Education						
No education	99	3360 (240, 6960) ^a^	31 (31.3)	39 (39.4)	29 (29.3)	4.47 (1.62, 12.33)
Primary to high school	148	5040 (2400, 9360) ^b^	67 (45.3)	64 (43.2)	17 (11.5)	1.40 (0.49, 3.99)
SSC or above	59	5040 (2400, 8880) ^b^	27 (45.8)	27 (45.8)	5 (8.5)	1.00 (reference)
Socioeconomic status						
Poor	92	4920 (2400, 9600) ^a^	41 (44.6)	36 (39.1)	15 (16.3)	1.00 (reference)
Middle class	214	4200 (1680, 8400) ^b^	83 (38.8)	94 (43.9)	37 (17.3)	1.37 (0.65, 2.86)
Occupation						
Farmer	66	7320 (3360, 13,320) ^a^	44 (61.1)	21 (29.2)	7 (9.7)	1.00 (reference)
Housewife	146	4200 (1440, 6960) ^b^	52 (35.6)	66 (45.2)	28 (19.2)	2.20 (0.91, 5.32)
Employees	53	2880 (480, 5280) ^b^	12 (22.6)	26 (49.1)	15 (28.3)	3.67 (1.37, 9.79)
Business	24	6480 (1680, 13,200) ^a^	12 (50)	12 (50)	0	-
Diabetes Status						
No diabetes	217	4560 (1200, 8640) ^a^	85 (39.2)	86 (39.6)	46 (21.2)	1.00 (reference)
Diabetes	41	3720 (840, 6720) ^b^	12 (29.3)	24 (58.5)	5 (12.2)	0.52 (0.18, 1.52)
Unknown	49	5520 (2400, 10,560) ^a^	28 (57.1)	20 (40.8)	1 (2)	-

^†^ without any subscript or subscript with same letter, e.g., “a” are for the nonsignificant difference. Different letters “a” and “b” indicates a significant difference at least at a 5% level of significance; ^‡^ OR (95% CI) adjusted for variables in model.

## Data Availability

The data presented in this study are available on reasonable request from the corresponding author. The data are not publicly available due to the study is ongoing for intervention.
